# Synthesis of Arylpiperazine-Based CNS Ligands: Batch Disconnections and Their Continuous-Flow Implementation

**DOI:** 10.3390/molecules31203682

**Published:** 2026-10-14

**Authors:** Daeun Jeong, Ji Myoung Han, Ji Hoo Lee, In Young Oh, Ye Rim Cho, Yong-Ju Kwon

**Affiliations:** Department of Chemistry, Seoul Women’s University, Seoul 01797, Republic of Korea; jdaeun2404@gmail.com (D.J.); ghw4197@swu.ac.kr (J.M.H.); jipori0315@swu.ac.kr (J.H.L.); oin0531@swu.ac.kr (I.Y.O.); esther1218@swu.ac.kr (Y.R.C.)

**Keywords:** arylpiperazine, CNS ligands, continuous-flow chemistry, batch synthesis, reductive amination, chemoselective hydrogenation, telescoped synthesis, process intensification, flow-oriented design, medicinal chemistry

## Abstract

The 1-arylpiperazine unit recurs throughout centrally acting drugs, particularly ligands of serotonin and dopamine receptors, where its modular architecture—aryl group, piperazine core, linker, terminal fragment—has made it a durable design template. Three principal intermodule disconnections—the aryl-nitrogen bond, the piperazine-linker bond, and the linker-terminal bond—provide an organizing framework for the routes reviewed here. This review compares representative batch routes with reported continuous-flow implementations, distinguishing related methodological precedents, individual transformations in drug-specific routes, and linked multistep sequences. Three observations emerge from the reviewed examples. First, the drug-directed flow routes examined use preformed arylpiperazine building blocks. Second, dechlorination reported during cariprazine reductive amination highlights the importance of catalyst and solvent selection for that substrate. Third, the reported flow implementations address selected process challenges, while the multistep examples highlight the importance of compatibility between reaction and work-up operations. These observations inform a practical framework for evaluating batch and flow options for individual routes.

## 1. Introduction

Drug discovery for the central nervous system (CNS) is among the most demanding areas of medicinal chemistry, as follows: a viable neurotherapeutic must reconcile potency and selectivity at the intended target, metabolic stability, an acceptable safety margin, and sufficient exposure across the blood–brain barrier, and these constraints frequently pull molecular design in opposing directions. For example, 4-arylazo pyrazole carboxamides showed potent cholinesterase inhibition in vitro but were computationally predicted not to cross the blood–brain barrier, illustrating the need to distinguish biochemical potency from CNS exposure [1]. Nitrogen-containing heterocycles have become a mainstay of this effort, since a basic nitrogen offers a tunable handle for salt formation and solubility, participates in hydrogen bonding, and forms the reinforced ionic contact with a conserved aspartate residue that anchors many ligands within aminergic receptor pockets [2]. Among these motifs, the arylpiperazine unit is unusually prominent in agents acting on serotonergic and dopaminergic systems. Its architecture—an aryl group, the piperazine core, a linker, and a terminal fragment—is inherently modular, and structure–activity work across the long-chain arylpiperazine (LCAP) class has shown that affinity, selectivity, and functional profile respond to each module independently [3,4,5]. That modularity has made the scaffold a durable design template and a recurring feature of marketed CNS drugs, from aripiprazole, cariprazine, and ziprasidone to buspirone, trazodone, vilazodone, vortioxetine, and flibanserin [6,7,8,9,10,11,12,13].

**Figure 1:**
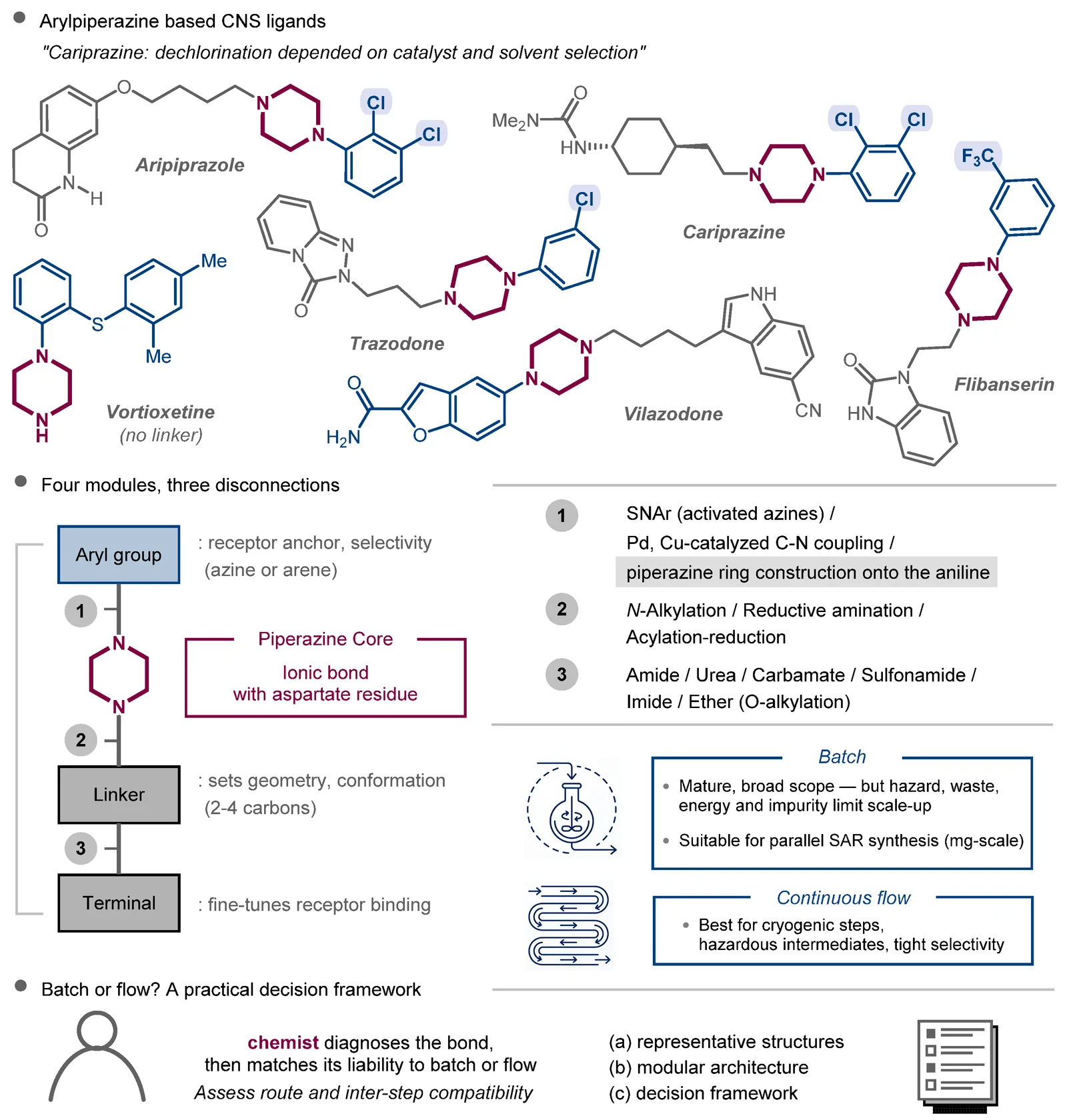
Overview of arylpiperazine-based CNS ligands: representative structures, modular architecture, and the batch-versus-flow decision framework.

Every one of these agents was developed, and most are still manufactured, in the stirred vessel. Pharmaceutical synthesis at large, however, has been reorienting away from it. Continuous processing offers efficient heat and mass transfer, precise control of residence time, safer handling of hazardous reagents and of forcing thermal regimes, and increased total production through longer operation without necessarily enlarging the reactor [14,15,16,17,18]. The supporting framework has matured alongside the chemistry, as follows: continuously manufactured products have been approved, and ICH Q13 now provides harmonized guidance for both drug substances and drug products [19], while uninterrupted end-to-end preparations have been demonstrated for a growing number of APIs, including arylpiperazine drug substances themselves [20,21,22]. For an established scaffold, then, the question is no longer whether its chemistry can be run continuously, but how far that conversion has been carried—and which parts of the synthesis it has reached (Figure 1).

What the transition has not been is uniform. Adoption has proceeded transformation by transformation rather than route by route, concentrating wherever a batch operation is uncomfortable to run rather than wherever a bond is important to make. The map of what has been implemented in flow is therefore not the map of what a scaffold’s synthesis consists of, and the existing literature does not permit the two to be compared, as follows: reviews of the arylpiperazine scaffold address pharmacology or solid-state structure [23], and where synthesis has been reviewed, it has been organized by approval data across the piperazine class as a whole rather than by the disconnections of one scaffold [24]; the flow literature, meanwhile, is organized around reaction types or process goals rather than around a therapeutic class. The arylpiperazine ligands are a particularly informative case for closing that gap. Several of the selected ligands contain halogenated or trifluoromethylated aryl groups. The cariprazine example discussed in Section 4.2 illustrates the importance of catalyst and solvent selection for controlling dechlorination [25]. The relevant flow literature is also small enough to be examined case by case, so each reported process can be set against the specific batch liability that motivated it. Pharmacological detail and receptor structural biology lie outside our scope.

Section 2 sets out the scaffold, the three disconnections its architecture defines, and the reason one of them is usually inherited rather than forged. Section 3 surveys batch practice along those bonds and locates the constraints it leaves behind—hazard, waste, energy demand, and impurity control. Section 4 examines the reported flow implementations, from related methodological precedents and individual transformations in drug-specific routes to linked multistep sequences, with attention to inter-step compatibility and the extent of implementation within the literature reviewed. Section 5 converts that map into criteria for deciding which steps to move into flow and which to leave in batch. The pattern that emerges is a consistent one, and the following is the argument of this review: continuous processing has advanced the reductive chemistry of this class, and has scarcely touched the assembly of the scaffold itself.

### Scope, Literature Search, and Evidence Classification

This narrative review focuses on selected synthetic routes to the arylpiperazine-based CNS ligands listed in Table 1 and their intermediates. Literature searches were conducted in Reaxys, Google Scholar, and Google Patents using combinations of individual drug names and arylpiperazine. The searches were completed on 31 August 2026. The synthetic literature discussed spans 1960–2026. Original research articles and patents describing relevant synthetic routes, reaction conditions, or continuous-flow implementations were included. Related flow studies on other substrates were included as precedents for the reaction classes and process-design considerations discussed here. Publications addressing only pharmacology or formulation were used as background references rather than as evidence of synthetic implementation.

Flow examples were classified as related methodological precedents, individual transformations in drug-specific routes, or linked multistep sequences, based on the reported substrates, products, and process descriptions. Drug-specific routes included documented precursors before introduction of the piperazine unit; supporting transformations were distinguished from steps directly forming the intermodule bonds. Multistep sequences were differentiated according to whether intermediate isolation or offline/at-line work-up interrupted the connection between flow modules. For patents, worked experimental examples were distinguished from general disclosures and claimed specifications. Statements that no implementation was identified refer only to the defined scope and literature search and do not establish intrinsic unsuitability for continuous processing.

## 2. The Arylpiperazine Scaffold in CNS Drugs

### 2.1. Representative CNS Drugs Containing Arylpiperazine Motifs

Table 1 collects representative marketed agents built on the 1-arylpiperazine motif. Rather than surveying these drugs individually—their pharmacology has been reviewed thoroughly elsewhere [2,3,23]—it is more useful here to sort them by the electronic character of the aryl group, because this single feature largely dictates how the aryl–nitrogen bond can be constructed.

A first group carries an electron-deficient heteroaryl group directly on the piperazine nitrogen. Buspirone, gepirone, and tandospirone bear a pyrimidin-2-yl group; ziprasidone and lurasidone a 1,2-benzisothiazol-3-yl group. The corresponding heteroaryl halides are sufficiently activated to undergo nucleophilic substitution with piperazine.

A second—and considerably larger—group carries a carbocyclic aryl bearing no activating substituent, as follows: the 2,3-dichlorophenyl group shared by aripiprazole and cariprazine, the 3-chlorophenyl of trazodone, the 3-(trifluoromethyl)phenyl of flibanserin, the benzofuran-5-yl of vilazodone, and the 2-(2,4-dimethylphenylsulfanyl)phenyl of vortioxetine. Halogen and trifluoromethyl substituents notwithstanding, none of these rings is activated toward addition–elimination in the manner of a nitroarene or an azine. For most marketed arylpiperazines, then, the aryl–N bond cannot be formed by nucleophilic aromatic substitution at all—a limitation whose synthetic consequences are taken up in Section 2.2.

For reductions involving halogenated substrates, halogen retention should be assessed under the intended reaction conditions. The cariprazine example discussed in Section 4.2 illustrates this consideration [25].

### 2.2. Structural Anatomy and Points of Diversification

**Figure 2:**
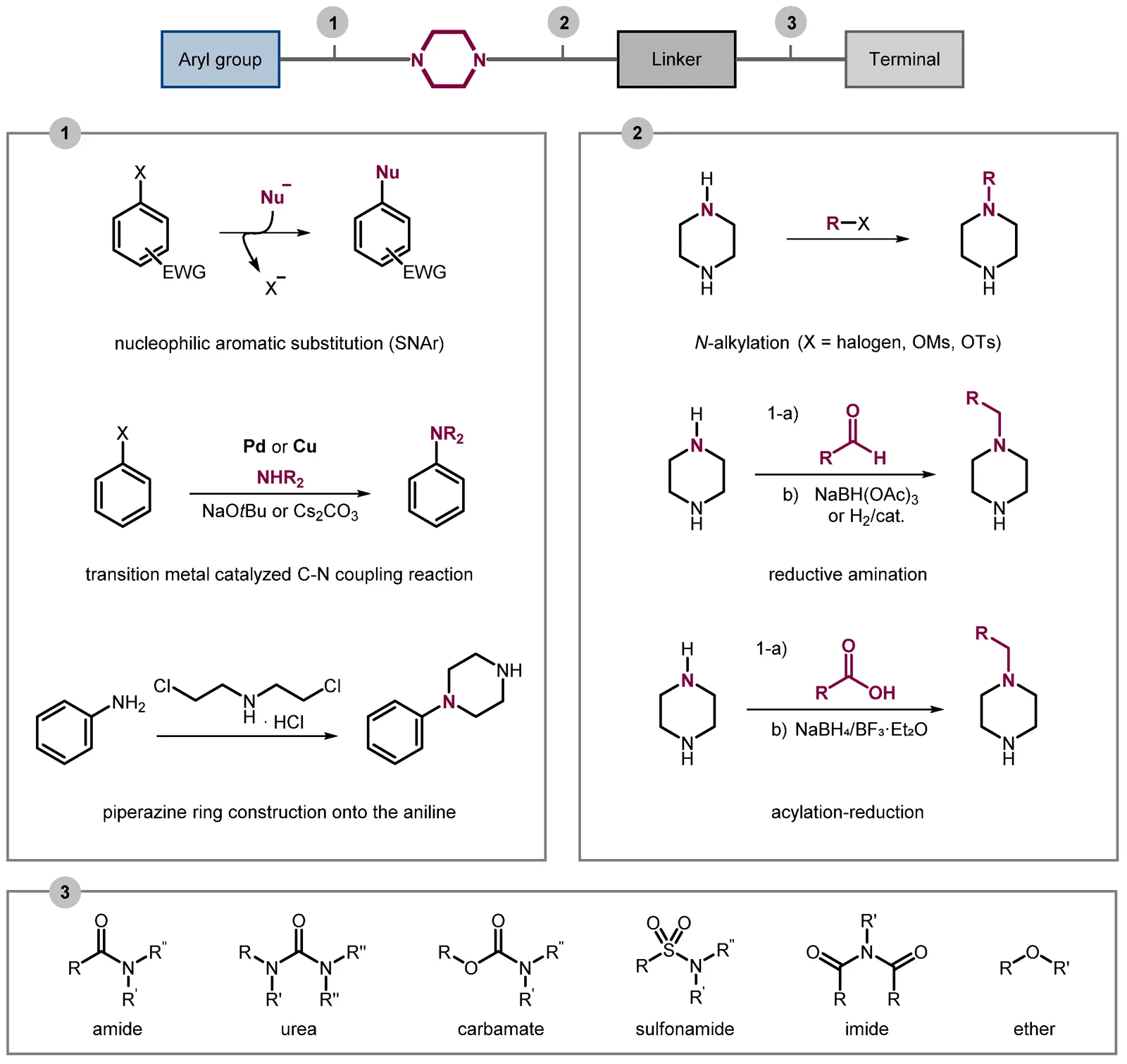
Reaction classes underlying the three arylpiperazine disconnections.

Structurally, these ligands decompose into four modules (Figure 2), as follows: the aryl group, the piperazine core, a linker, and a terminal fragment. Where the linker is an alkyl chain of two to four carbons terminating in a heterocyclic amide or imide, the resulting compounds are conventionally described as long-chain arylpiperazines (LCAPs), one of the largest classes of serotonin and dopamine receptor ligands [2,3,4,5]. Affinity, selectivity, and functional profile respond to all three variable modules—the aryl substituent, the length and preferred conformation of the linker, and the terminal group—which is precisely why the scaffold has proved so durable as a design template [2,3,4,5]. Vortioxetine is the instructive exception, as follows: its piperazine is joined directly to a 2-(arylsulfanyl)phenyl group with no linker or terminal fragment at all, a reminder that the modular template describes a strong tendency rather than a rule.

Read synthetically, the same four modules define three disconnections, as follows:

(a) the aryl–*N*(piperazine) bond
(b) the piperazine–linker bond
(c) the linker–terminal bond

Disconnection (a) admits three approaches whose applicability is set by the aryl group’s electronics, as follows: SNAr for the activated heteroaryl halides of Section 2.1, transition-metal-catalysed C–N coupling for unactivated arenes, and construction of the piperazine ring onto the corresponding aniline. Disconnection (b) can be addressed by *N*-alkylation or reductive amination at the free piperazine nitrogen, or by *N*-acylation followed by amide reduction. Disconnection (c) concerns the connection between the linker and terminal fragment and may involve acyl or sulfonyl transfer, *N*- or *O*-alkylation, reductive amination, or C–C bond formation, depending on the route. The structural anatomy of the scaffold thus maps directly onto a small and recurring set of synthetic transformations—but not onto an even distribution of synthetic effort.

The unevenness is the point, and it originates at disconnection (a). Because SNAr is unavailable for the larger of the two aryl classes, one reported approach is not to form the aryl–N bond at all but to build the piperazine ring onto the aniline, as in the cyclization of 2,3-dichloroaniline with bis(2-chloroethyl)amine hydrochloride at 120–220 °C that supplies 1-(2,3-dichlorophenyl)piperazine, the common building block of both aripiprazole and cariprazine [24,30]. Variants of this transformation, performed at scale by suppliers, populate the catalogues from which arylpiperazine fragments are now bought. The fragment therefore enters most medicinal chemistry campaigns pre-formed, and disconnection (a) is bypassed entirely rather than exercised—a point easily overlooked in retrosynthetic discussions of the scaffold, since (a) is the bond most prominent in the methodological literature.

What follows is that disconnections (b) and (c) carry nearly all of the analogue-generating work, and the same imbalance is visible in the continuous-flow literature. Reported processes include the reductive amination at (b) and the urea formation at (c) [25,31], as well as *N*-alkylation in trazodone routes at (b) or (c), depending on the order of fragment assembly (Section 4.2). In these routes, the aryl–*N* bond is inherited from a pre-formed building block. Section 3 examines batch practice along all three bonds and identifies the liabilities it leaves behind; Section 4 then asks how far continuous implementation has actually advanced, and Section 5 turns the answer into criteria for choosing between the two.

## 3. Batch Strategies Toward Arylpiperazine-Based CNS Ligands

### 3.1. Disconnection 1: The Aryl–N(piperazine) Bond

**Scheme 1:**
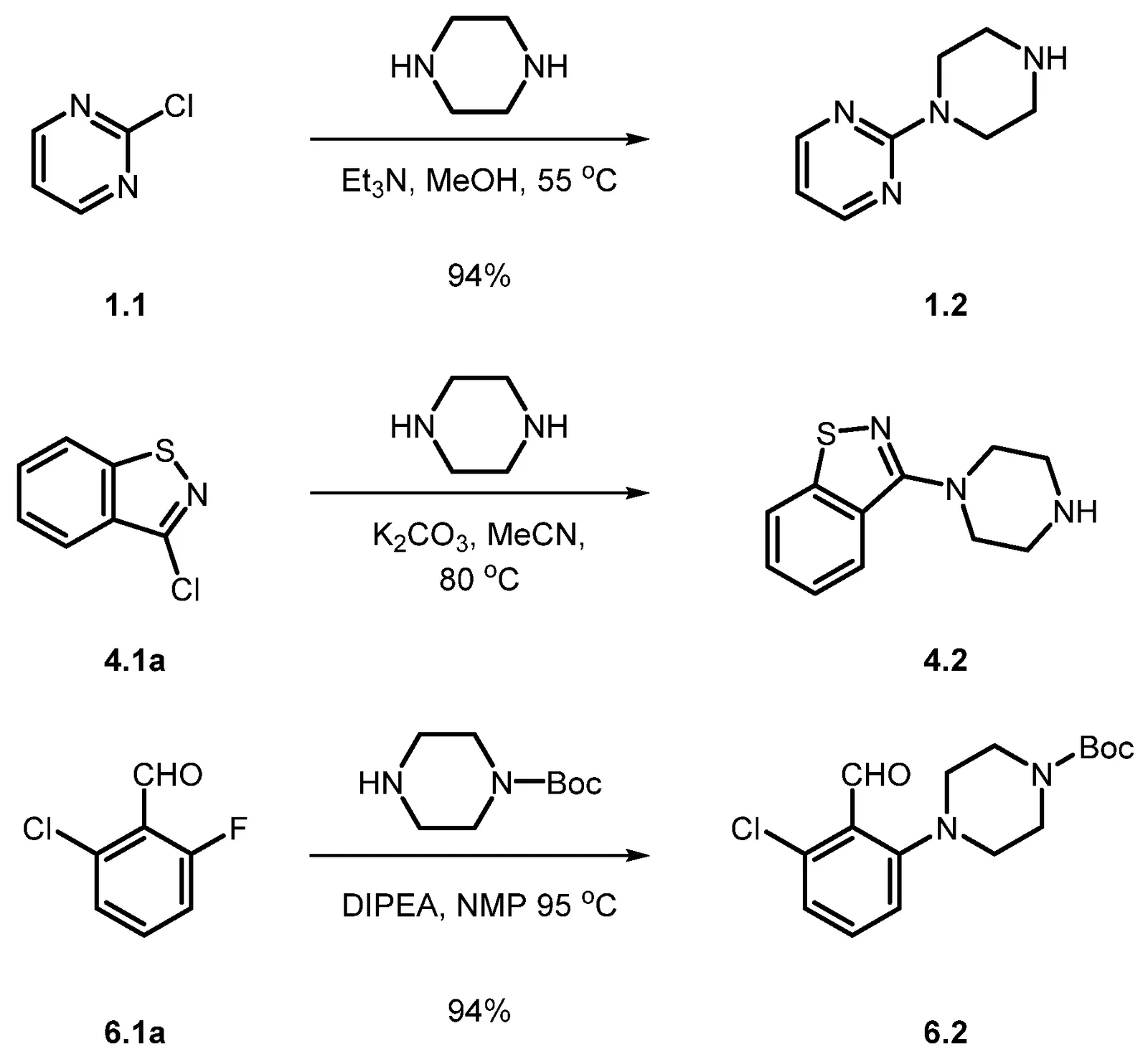
SNAr routes to arylpiperazines from activated halides.

***Activated rings: substitution needs no catalyst.*** Where the aryl group is an activated heteroaryl group, disconnection (a) is served directly by nucleophilic aromatic substitution. The pyrimidin-2-yl group of buspirone [32] and the 1,2-benzisothiazol-3-yl group of ziprasidone [33,34] are both installed by displacement of a halide from the activated ring carbon (Scheme 1). Conditions are undemanding, as follows: piperazine in excess, with or without K_2_CO_3_ or DIPEA, in MeCN, DMF, or *n*-butanol at 80–140 °C over 12–24 h [33,34]. Because piperazine is symmetric and bis-nucleophilic, mono-arylation is normally secured by using the amine in two- to five-fold excess; mono-Boc-piperazine followed by acidic deprotection gives a cleaner profile [35] at the cost of two additional operations, and is reserved for cases where the piperazine is precious. Activation need not come from the ring nitrogen alone—an *ortho*-carbonyl group suffices, and the fluoride of 2-chloro-6-fluorobenzaldehyde is displaced by *N*-Boc-piperazine in 94% yield (**6.2**) [35,36,37], the aldehyde then serving as the handle for the annulation described below.

***Unactivated rings: build a ring instead of forging a bond.*** For the carbocyclic aryls that predominate among marketed agents, one reported approach is not to form the aryl–N bond at all but to build the piperazine ring onto the corresponding aniline. Cyclization of 2,3-dichloroaniline with bis(2-chloroethyl)amine hydrochloride [30]—or with diethanolamine under concentrated HCl, which generates the same bis-electrophile in situ (**7.2**) [38]—at 120–220 °C delivers 1-(2,3-dichlorophenyl)piperazine. The same disconnection serves substrates far removed from that fragment, as follows: the nitrobenzofuran **11.1** is first reduced with H_2_ over Pd/C in MeOH, and the resulting aniline is subsequently cyclized with bis(2-chloroethyl)amine hydrochloride in refluxing butanol [39]. The diarylsulfide aniline is converted to vortioxetine hydrochloride by reaction with bis(2-chloroethyl)amine hydrochloride in methyldiglycol at 130 °C for three days, affording the hydrochloride salt in 53% yield [40]. The route is unlovely—forcing temperatures, a bis-electrophilic reagent of some toxicity, moderate yields, and appreciable acidic waste—but it is cheap, requires no catalyst, and runs on abundant anilines.

**Scheme 2:**
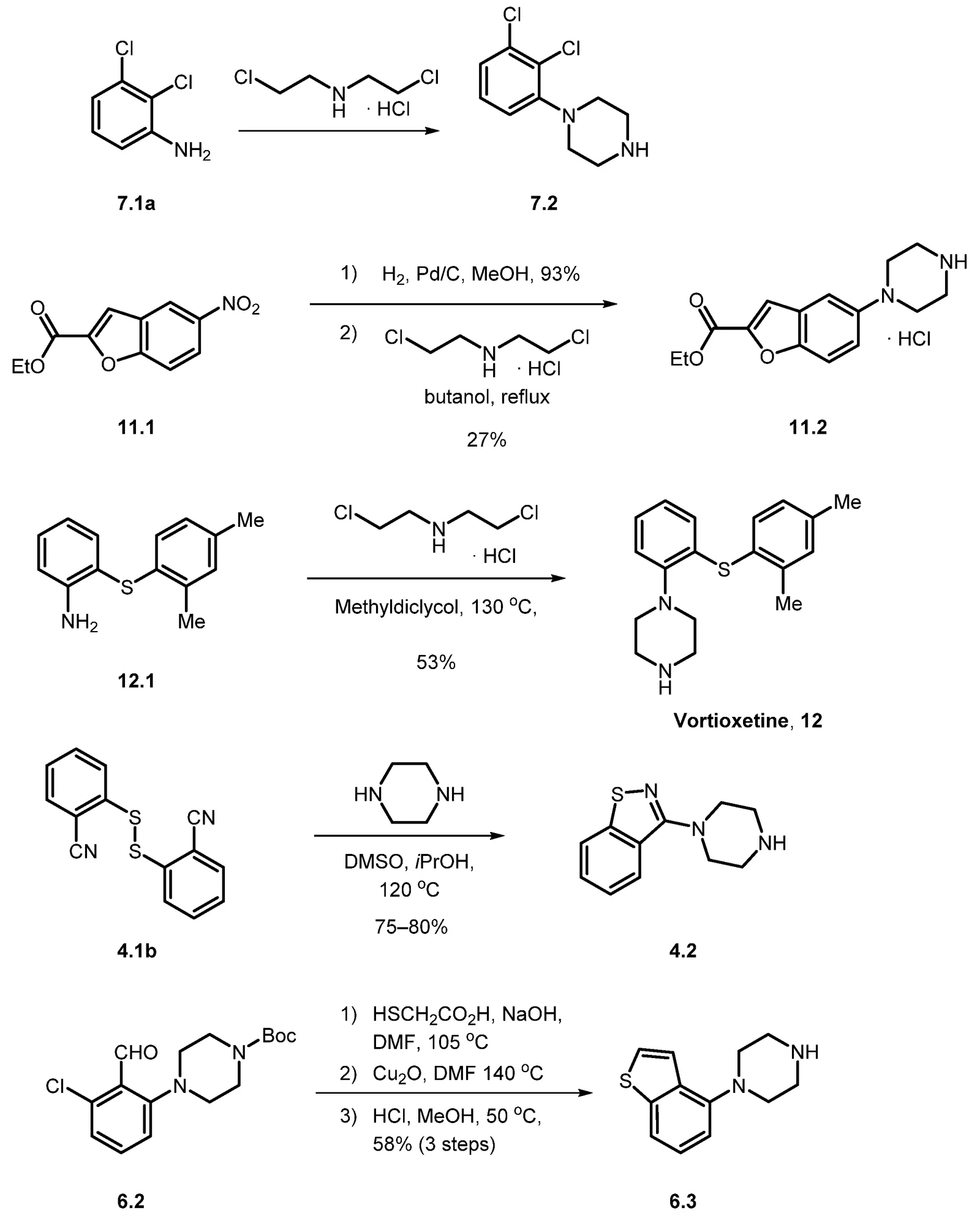
Routes in which the aryl–N bond arrives with a ring rather than a coupling.

Heteroaromatic ring construction provides a further alternative. Reaction of 2,2′-dithiobis(benzonitrile) with piperazine affords the benzisothiazole derivative **4.2** used in ziprasidone and lurasidone synthesis [41]. By contrast, aldehyde **6.2** is elaborated to the benzothiophene derivative **6.3** used in brexpiprazole synthesis, retaining the pre-existing aryl–N bond [35,36,37] (Scheme 2).

**Scheme 3:**
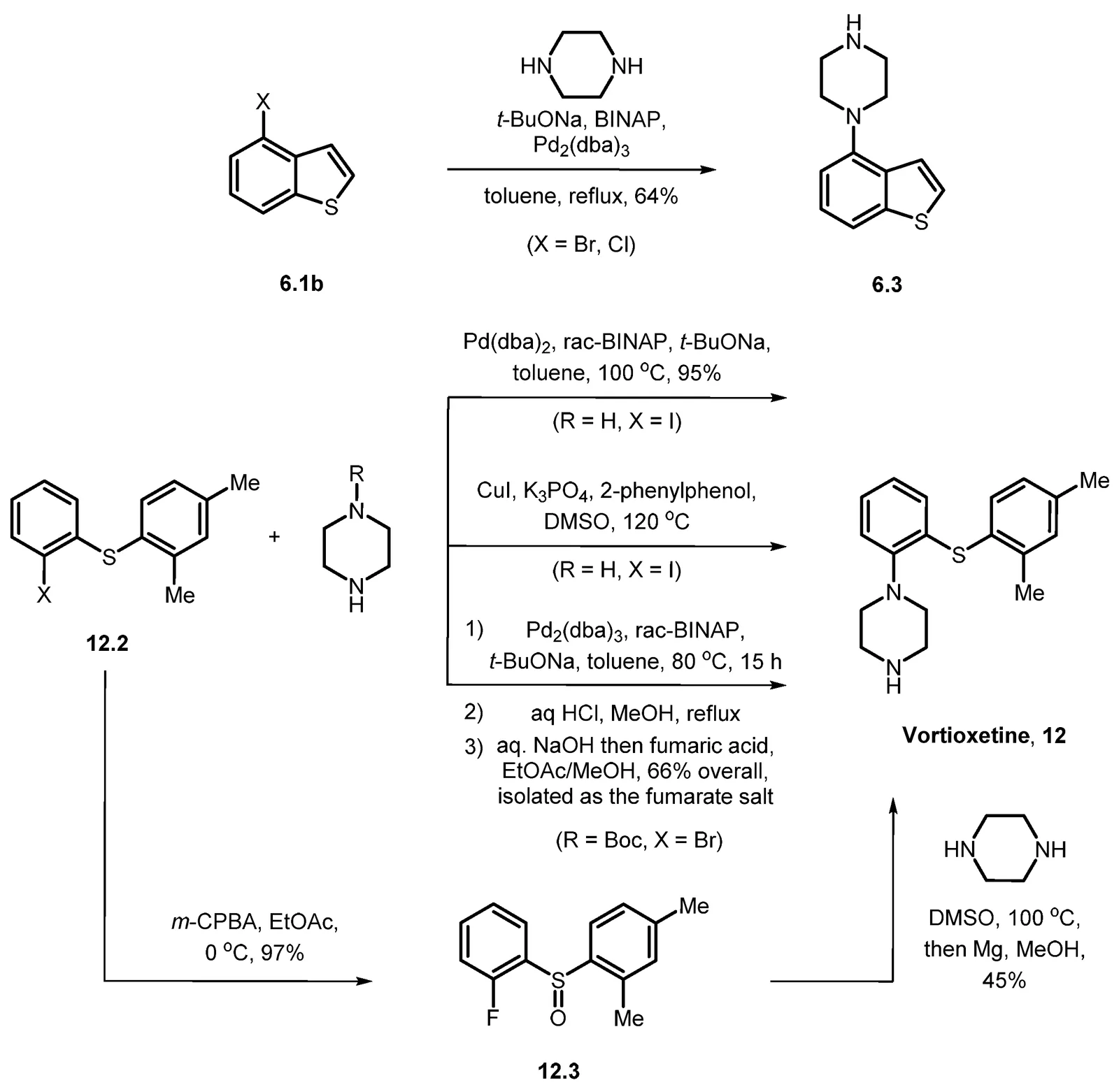
Metal-catalysed and metal-free routes to arylpiperazines.

***Metal-catalysed amination: indispensable, but narrow.*** Palladium catalysis becomes necessary in the narrow case where the aryl is unactivated and the arylpiperazine is not available as a fragment. The benzothiophene fragment of brexpiprazole is accessible by Buchwald–Hartwig amination (Scheme 3) [36]. A related 2,3-dichlorophenyl amination reported in 75% yield affords a seven-membered diazepane hydrochloride, rather than the six-membered piperazine building block required for cariprazine [42]. Vortioxetine is different, as follows: carrying no linker, the arylpiperazine is not a precursor to the drug but the drug itself, so the aryl–N bond cannot be inherited from a purchased fragment. More generally, dialkylbiaryl phosphines such as RuPhos and BrettPhos have expanded the scope of Buchwald–Hartwig amination, with ligand selection depending on the amine and aryl electrophile [43]. Both unprotected and *N*-Boc-protected piperazine have been used in vortioxetine synthesis. The unprotected route affords vortioxetine in 95% yield [44], whereas the Boc-protected route gives the fumarate salt in 66% overall yield after coupling, deprotection, and salt formation [45]. These values reflect different synthetic sequences and isolation forms and do not establish a yield penalty attributable to Boc protection alone. The coupling is robust enough to proceed under mechanochemical ball-milling without precautions against air or moisture including Boc-protected vortioxetine itself in 69% [46].

The limitations are economic and regulatory rather than chemical. Palladium is orders of magnitude more expensive than base metals, and residual palladium in an API is tightly specified, imposing scavenging operations downstream. These pressures have driven copper-catalysed alternatives, as follows: CuI with K_3_PO_4_ and 2-phenylphenol effects the same vortioxetine coupling [44], and a recent one-pot process forms both the C–S and C–N bonds under copper catalysis in a single operation, its authors citing palladium’s cost explicitly [47]. Oxidation to the sulfoxide enables nucleophilic aromatic substitution with piperazine. The patent reports 71% yield for this substitution and 64% for subsequent Mg/I_2_-mediated reduction, corresponding to a calculated combined yield of approximately 45% for the two separately reported steps [48]. A further constraint, the heterogeneity of the inorganic base, is mundane in a flask but returns in Section 4 as the principal obstacle to running this chemistry continuously.

### 3.2. Disconnection 2: The Piperazine–Linker Bond

**Scheme 4:**
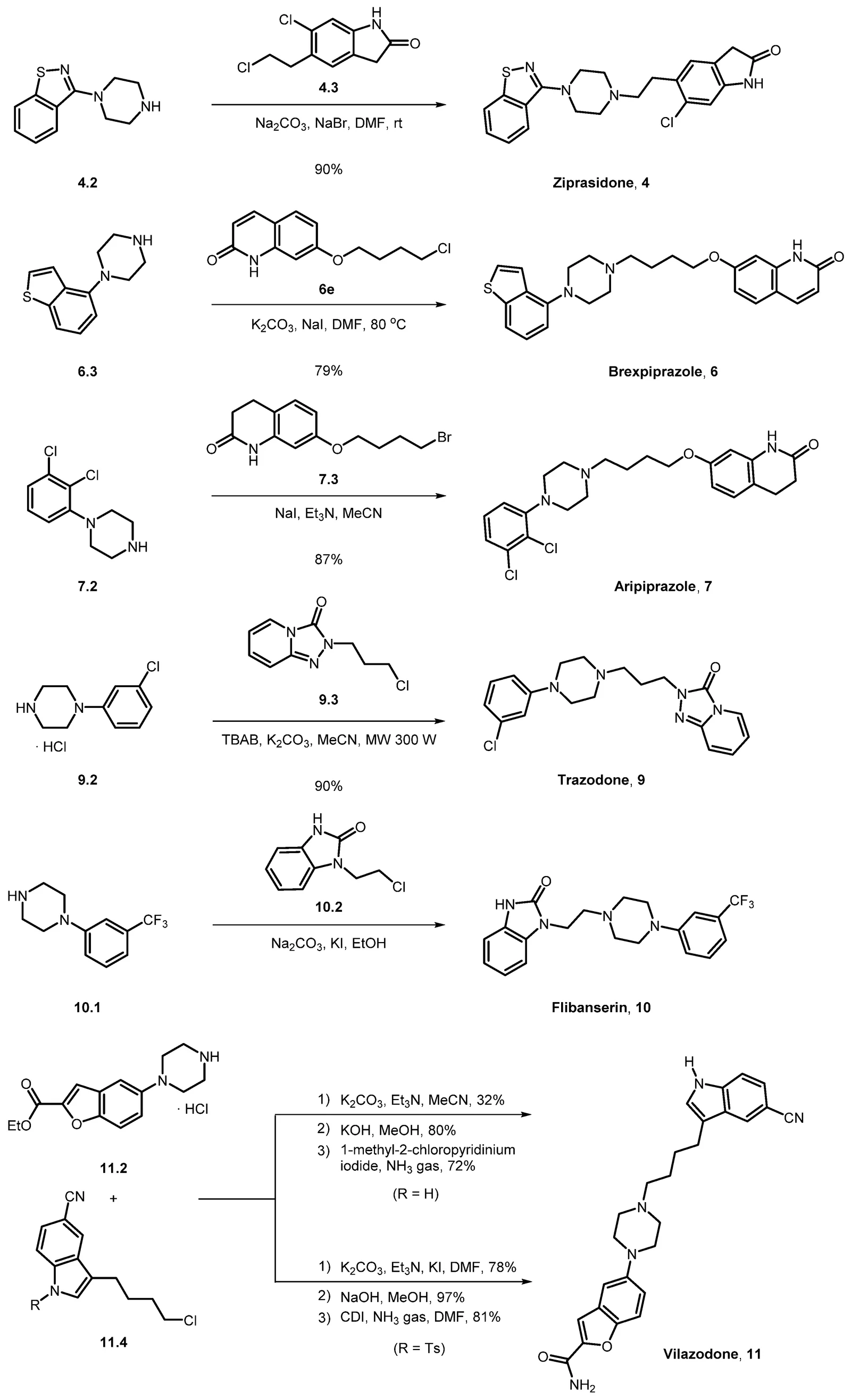
*N*-Alkylation routes to the linker.

***Alkylation with activated halides: the workhorse.*** Disconnection (b) is where the bulk of arylpiperazine synthesis actually occurs, and *N*-alkylation is its workhorse. The free piperazine nitrogen of a purchased fragment is a competent nucleophile, and the ω-haloalkyl electrophiles bearing the terminal pharmacophore are convergent partners (Scheme 4). Aripiprazole is the canonical case, as follows: 1-(2,3-dichlorophenyl)piperazine and 7-(4-bromobutoxy)-3,4-dihydroquinolin-2(1*H*)-one are united with K_2_CO_3_ in refluxing acetonitrile [49]. Ziprasidone [33,34], brexpiprazole [36], trazodone [50], flibanserin [51], and vilazodone [39] are assembled by the same logic, differing only in the length of the tether—two, three, or four carbons—and in the heterocycle it carries. Conditions are correspondingly standard, as follows: near-stoichiometric electrophile and arylpiperazine, K_2_CO_3_ or an amine base, catalytic iodide, MeCN or DMF, reflux for 8–24 h.

One detail recurs so consistently that it is easily mistaken for incidental. Five of the six couplings in Scheme 4 employ an iodide or bromide additive, and in each the substrate delivered is a chloride, as follows: the halide is exchanged in situ, so that the cheap electrophile is bought while the reactive one is generated only as needed. Where the substrate is already a bromide, as in aripiprazole, the additive is retained nonetheless. This in situ Finkelstein is the closest thing the transformation has to a signature.

Two variations are worth noting. Trazodone is coupled under microwave irradiation with tetrabutylammonium bromide as phase-transfer catalyst, both measures addressing the same difficulty—the inorganic base is insoluble in acetonitrile and the reaction is heterogeneous throughout, a point that returns in Section 4. And in vilazodone, alkylation of the indole-bearing electrophile proceeds in 32% with the NH free but 78% when it is tosylated [39,52]; the competing nitrogen must be masked, exactly as *N*-Boc-piperazine is masked in Section 3.1.

Three liabilities attend the method, as follows: over-alkylation at the newly tertiary nitrogen affords quaternary ammonium salts; elimination competes at elevated temperature; and residual alkyl-halide reagents may require assessment and control as potentially mutagenic impurities under ICH M7 [53]. None impedes the assembly of an analogue array on milligram scale—which is why *N*-alkylation remains unrivalled for library work—but all become expensive at scale, and it is this asymmetry, rather than any deficiency of scope or yield, that motivates the alternatives below.

**Scheme 5:**
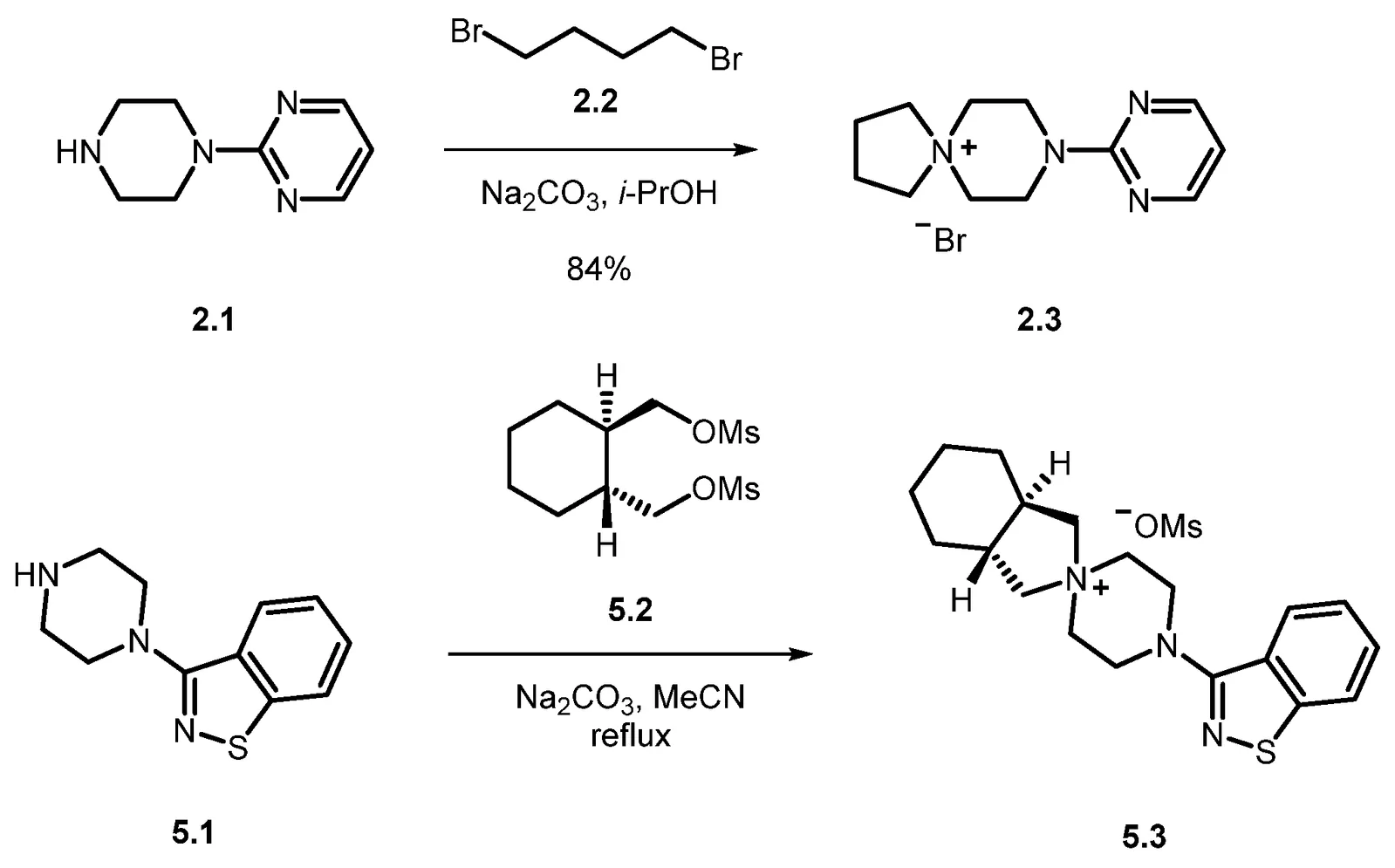
Azoniaspiro salts as linker carriers.

***Two alkylations at once: the azaspiro salt.*** The first liability can be inverted. Treating the arylpiperazine with a 1,4-bis-electrophile rather than a monofunctional one sequentially alkylates the initially free piperazine nitrogen twice to form an azoniaspiro salt, and the product is not an impurity but a crystalline azoniaspiro salt (Scheme 5): 1-(2-pyrimidinyl)piperazine and 1,4-dibromobutane give the spiro bromide **2.3** in 84% [54], the benzisothiazolylpiperazine **5.1** and a cyclohexane-derived dimesylate give **5.3** in 90% [55]. The salts are carriers rather than products—attack by an imide anion at a ring carbon opens the quaternary centre regioselectively, installing linker and terminal group in one operation (Section 3.3). The tactic buys the following three things: the symmetry of piperazine becomes an asset rather than a hazard, the intermediates are filterable solids in place of oils, and the alkylating agent is consumed well upstream of the final substance. Its scope is correspondingly narrow—it requires a terminal nucleophile acidic enough to be deprotonated and a linker short enough to close a five- or six-membered ring—but within that scope it is a reported synthetic option.

***Reductive amination and acylation–reduction.*** Reductive amination forges the same bond from a carbonyl rather than a halide, and is the standard response to the impurity burden just described. An aldehyde bearing the nascent linker is condensed with the arylpiperazine and reduced in situ, most often with NaBH(OAc)_3_ in DCM or THF at ambient temperature. This avoids the use of alkyl-halide electrophiles and their associated impurity-control burden, although other potential mutagenic impurities still require assessment under ICH M7.

**Scheme 6:**
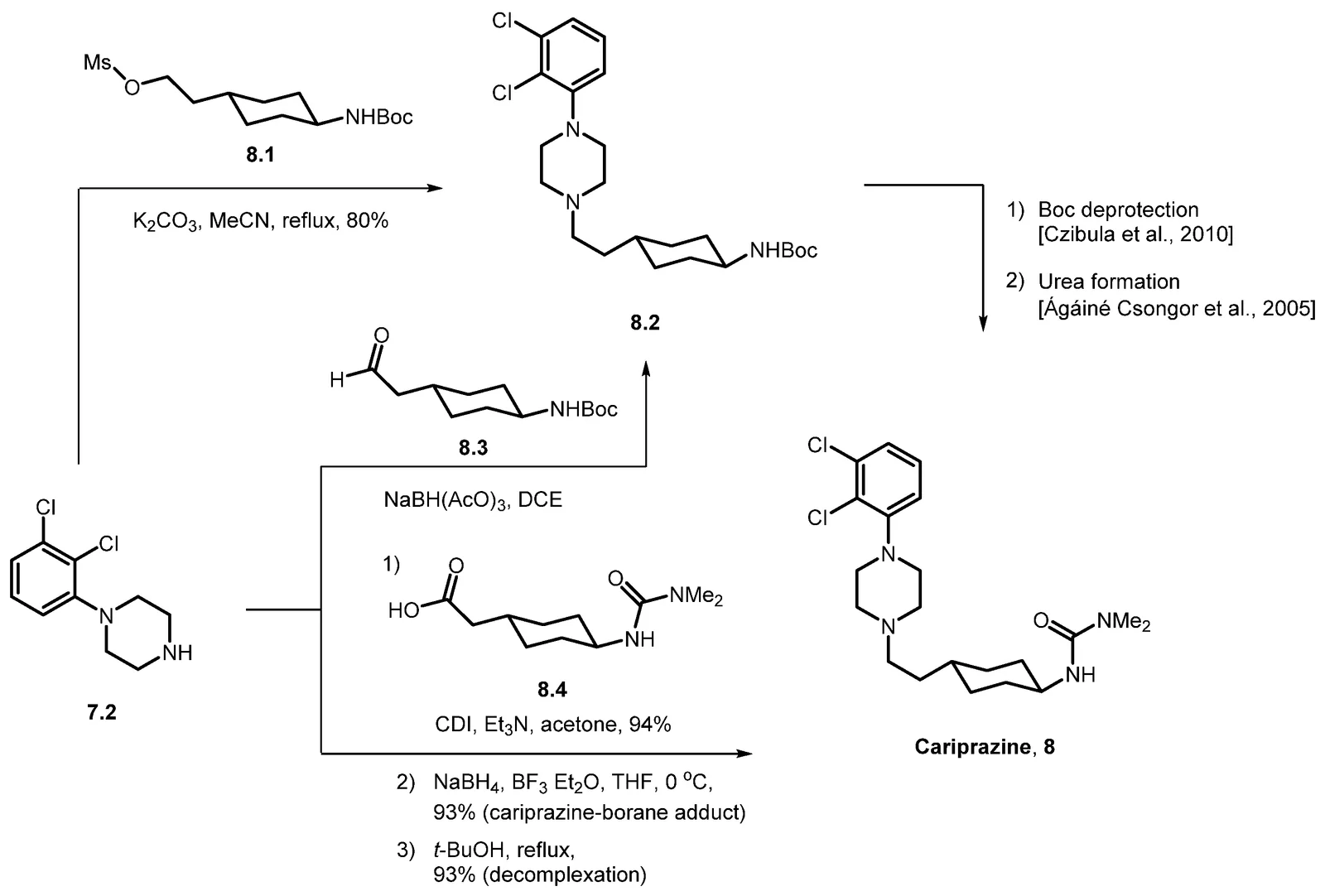
*N*-Alkylation, reductive amination, and acylation–reduction routes to cariprazine [56,42].

Cariprazine is the pertinent example, and an unusually clean one, because all three tactics have been reduced to practice on the same bond (Scheme 6). Alkylation of **7.2** with the mesylate **8.1** gives the Boc-protected intermediate **8.2** in 80% [56]. Reductive amination with the corresponding aldehyde also provides the Boc-protected intermediate using sodium triacetoxyborohydride in 1,2-dichloroethane [57]. The patent lists the 2,3-dichlorophenyl derivative among compounds prepared analogously, without specifying an isolated yield for that analogue. The mesylate route may entail additional assessment and control of residual alkylating impurities under ICH M7 [53]. A third option uses carboxylic acid 8.4, which already contains the dimethylurea terminal group. CDI-mediated coupling with the arylpiperazine in acetone affords the amide in 94.4% yield. Reduction with NaBH_4_/BF_3_·Et_2_O gives a cariprazine–borane adduct in 92.8% yield, followed by decomplexation in refluxing tert-butanol to afford cariprazine in 93% yield [58]. This terminal-first route avoids Boc protection at the coupling stage but requires a separate decomplexation operation.

The trade is not free. Stoichiometric hydride generates boron waste; NaBH(OAc)_3_ is moisture-sensitive, and DCM, its preferred medium, is a Class 2 solvent discouraged on scale; BF_3_·Et_2_O carries its own handling and quench penalties. More fundamentally, the aldehyde must come from somewhere—in the cariprazine case, from partial reduction of an ester with DIBAL-H, a transformation conventionally requiring cryogenic temperature to arrest over-reduction. Reductive amination, in other words, removes an alkyl halide and imports a cryogenic reduction in its place. Section 4 examines what happens when that exchange is renegotiated in flow.

### 3.3. Disconnection 3: The Linker–Terminal Bond

**Scheme 7:**
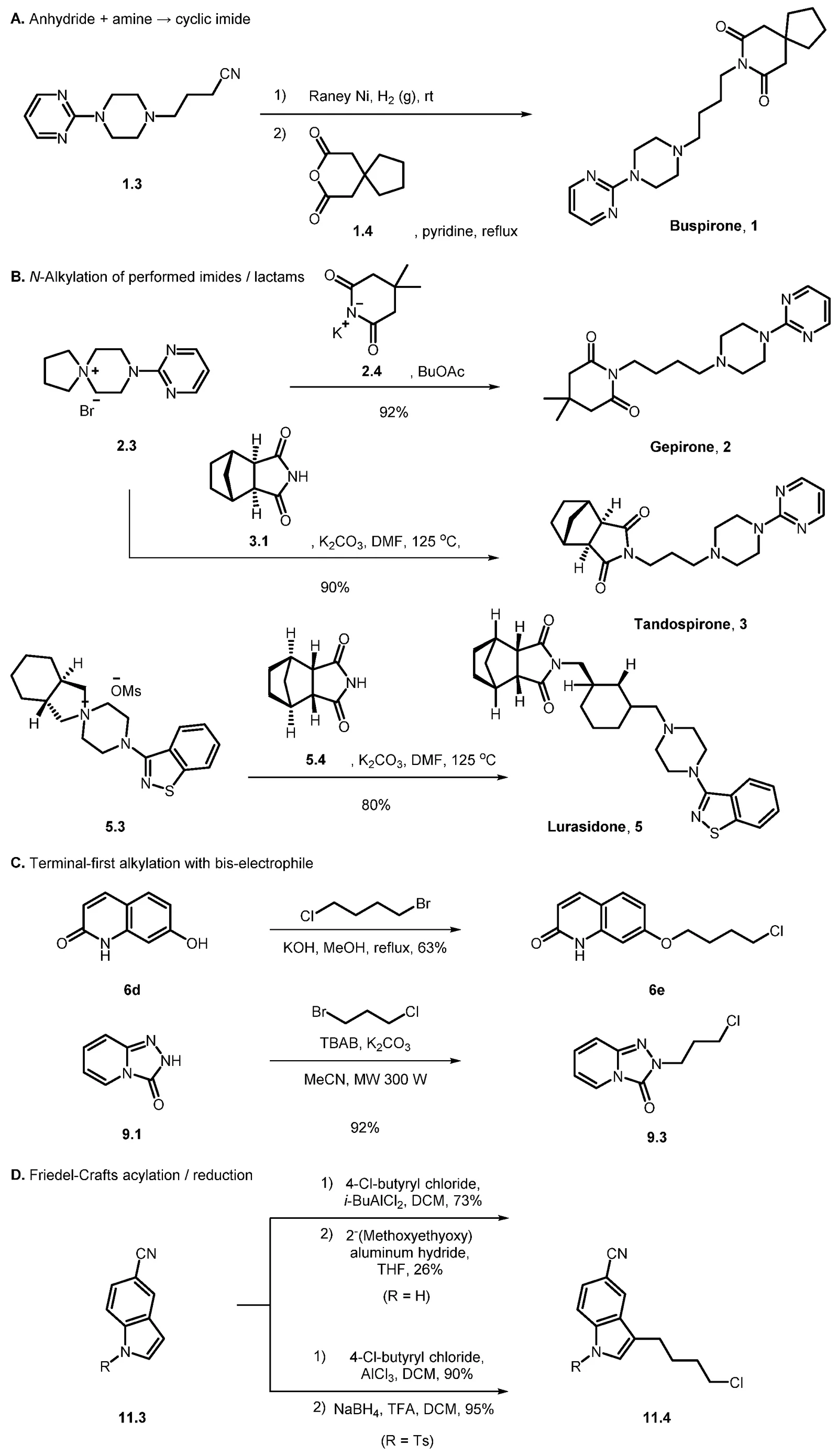
Assembly of the linker-terminal bond.

Disconnection (c) attaches the terminal fragment, and it runs in either of two polarities. Where the terminal is the electrophile, the bond is formed by acyl or sulfonyl transfer; where the terminal carries an N–H or O–H, it is the nucleophile and the linker arrives as an alkyl halide or sulfonate (Scheme 7). The choice is rarely free—it follows from which oxidation state the terminal’s own synthesis delivers, and from whether the linker has already been joined to the arylpiperazine.

***Acylation.*** The classical amide, carbamate, and sulfonamide terminals are formed with HATU, HBTU, or EDC and DIPEA in DMF or DCM at ambient temperature, or through the acid chloride, chloroformate, or sulfonyl chloride where the substrate permits. The azaspirodecanedione of buspirone is the same chemistry run twice (Scheme 7A), as follows: hydrogenation of nitrile **1.3** over Raney nickel unmasks a primary amine, which condenses with anhydride **1.4** in refluxing pyridine and closes to the imide in a single operation.

***Alkylation of a preformed terminal.*** When the imide is carried in preformed, the polarity inverts (Scheme 7B). Gepirone illustrates the mildest case, as follows: the potassium salt of **2.4** opens the pyrrolidinium ring of the azoniaspiro salt **2.3** in butyl acetate at 92%, the neutral tertiary amine serving as leaving group and the preformed salt removing any need for external base [59]. Tandospirone is obtained by nucleophilic ring opening of azoniaspiro salt **2.3** with imide **3.1** using K_2_CO_3_ in DMF at 125 °C, with a reported yield of 90% [60]. Similarly, reaction of azoniaspiro salt **5.3** with imide **5.4** using K_2_CO_3_ in DMF at 125 °C for 24 h affords lurasidone-free base in 80% yield [61].

***Terminal-first assembly.*** The terminal may equally be elaborated before the arylpiperazine is anywhere in the molecule, in which case a bis-electrophile does the work and chemoselectivity between its two ends carries the strategy (Scheme 7C), as follows: 1-bromo-4-chlorobutane alkylates the phenol of **6d** at bromine alone (KOH, refluxing MeOH, 63%), and 1-bromo-3-chloropropane alkylates the triazolopyridinone nitrogen of **9.1** (TBAB, K_2_CO_3_, microwave, 92%). In both, the surviving chloride is precisely the electrophile that disconnection 2 requires; these routes are therefore not an alternative to Scheme 7B so much as a reordering of the same two bond formations, chosen when the terminal is the cheaper fragment to elaborate. The C–C variant obeys the same logic (Scheme 7D), as follows: Friedel–Crafts acylation of cyanoindole **11.3** with 4-chlorobutyryl chloride installs the full linker in one step, after which the ketone must be removed—the free indole acylates in 73% but resists reduction (26%), whereas the *N*-tosyl substrate acylates in 90% and reduces in 95% with NaBH_4_ in TFA.

***Ureas.*** The terminal 3,3-dimethylurea of cariprazine is installed on a *trans*-4-aminocyclohexyl group by this disconnection, and three routes compete, as follows: formation of the cyclohexyl isocyanate followed by capture with dimethylamine, which is clean and rapid; CDI activation followed by amine addition, which avoids isolating the isocyanate at the price of an imidazole by-product; or acylation with a carbamoyl chloride. Isocyanates and carbamoyl chlorides can present handling hazards, depending on the reagent and operating conditions. These hazards are distinct from mutagenic-impurity concerns under ICH M7; structural alerts require compound-specific assessment and do not by themselves establish mutagenicity [53]. In situ generation and consumption in flow may reduce hazardous reagent inventory.

### 3.4. Summary: What Constrains Batch Synthesis

Read across Section 3.1, Section 3.2 and Section 3.3, the batch toolkit presents a consistent picture. The chemistry is mature, as follows: the disconnections are few, the scope is broad, the yields are serviceable, and nowhere above does a target fail to be made.

Two patterns nonetheless recur. The first is that protection is an operations-versus-yield calculation settled anew by each substrate, not a reflex. For vortioxetine, the protected and unprotected routes differ in reaction conditions and isolation form, so their reported yields do not establish a yield penalty attributable to Boc protection alone [44,45]. For vilazodone, *N*-tosyl protection is associated with higher reported yields in the alkylation and the reduction following Friedel–Crafts acylation [39,52]. The second, and more telling, is that where one bond can be built by several routes, yield is rarely what decides. For cariprazine, direct comparison of the reported yields is limited by the absence of an isolated yield for the relevant reductive-amination analogue [57] and by the separate decomplexation required in the acylation–reduction route [58]. Route selection must therefore account for precursor preparation, impurity-control requirements, and downstream operations. Selection among reduced-to-practice options is governed by what must be avoided, not by what can be achieved.

What must be avoided sorts into four categories:

- **Hazard.** Bis(2-chloroethyl)amine at 120–220 °C; handling of isocyanates and carbamoyl chlorides; BF_3_·Et_2_O and DIBAL-H and their quench penalties.
- **Waste.** Two- to five-fold excess piperazine; stoichiometric hydride and the corresponding boron burden; concentrated acidic effluent from ring construction; palladium scavenging downstream of any coupling.
- **Energy.** Eight to twenty-four hours at reflux for routine alkylations, 120–220 °C for the cyclizations, −78 °C for partial ester reduction—so that a single sequence may demand both thermal extremes.
- **Impurity control.** Assessment of residual alkyl halides, mesylate esters, and tosylate esters under ICH M7; residual metal specifications; quaternary ammonium salts and elimination products from over-alkylation.

The practical significance of these constraints depends on the target, production scale, and operating conditions. Section 4 examines the flow examples identified in this review by implementation level, with attention to the constraints addressed and to inter-step compatibility.

## 4. Continuous-Flow Implementation: How Far Has It Advanced?

Continuous processing addresses the four liabilities on which Section 3 closed, and does so directly, as follows: a small, thermally well-regulated channel improves heat and mass transfer, permits hazardous species to be generated and consumed without accumulation, brings forcing thermal regimes within safe reach, and replaces scale-up by scale-out [14,15,16,17,18]. Whether those advantages have been realized for this scaffold is a separate question, and the answer depends on what one counts as an implementation. This section therefore reads the literature by degree of advance rather than by bond. Section 4.1 collects the reaction classes relevant to arylpiperazine synthesis that have been demonstrated in flow on model substrates, where the chemistry transfers but the compounds are not ours; Section 4.2 turns to individual transformations in drug-specific routes; Section 4.3 to the two sequences in which such steps have been joined end to end—the stage at which the character of continuous processing is decided, and at which it most often fails. Section 4.4 reads the accumulated coverage back onto the three disconnections, where the pattern of what has been attempted, and what has not, becomes legible.

### 4.1. Reaction Classes in Flow: Demonstrations on Model Substrates

The transformations of Section 3 have all been run continuously somewhere, but rarely on the compounds of Table 1. This subsection establishes what is chemically available before Section 4.2 asks what has actually been applied.

**Scheme 8:**
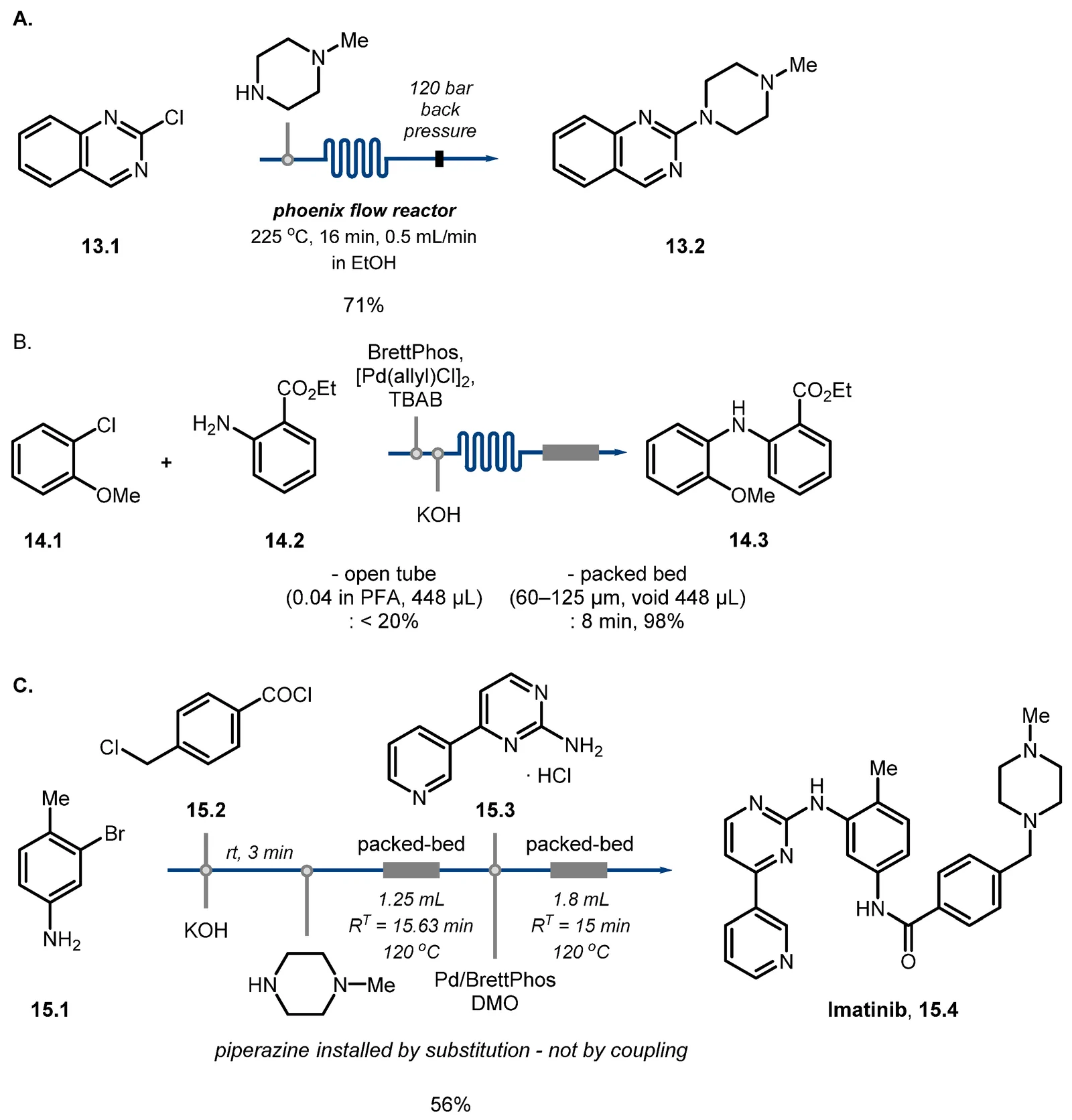
Reaction classes demonstrated in flow on model substrates: superheated SNAr (**A**), packed-bed Pd-catalysed C–N coupling (**B**), and the three-step imatinib synthesis, in which the piperazine is installed by substitution rather than coupling (**C**).

***Activated substitution transfers readily.*** The batch conditions of Section 3.1—80–140 °C for 12–24 h—describe precisely the regime in which continuous reactors are most comfortable, since a back-pressure regulator permits operation far above a solvent’s boiling point in an ordinary coil. The superheated protocol of Charaschanya and co-workers extends to piperazine itself, as follows: 2-chloroquinazoline **13.1** and 1-methylpiperazine give **13.2** in 71% at 225 °C under 120 bar with a 16-min residence time (Scheme 8A) [62]. One obstacle specific to this scaffold remains untreated, as follows: piperazine is bis-nucleophilic, and the batch answers to mono-arylation become a standing feedstock cost or an additional module in continuous operation.

***Metal-catalysed coupling: the obstacle is not the chemistry.*** For unactivated arenes, the difficulty in flow is neither yield nor scope but the stoichiometric inorganic base, whose salts precipitate in narrow channels and foul the reactor. Naber and Buchwald answered this with a biphasic toluene–aqueous KOH system in which TBAB shuttles hydroxide into the organic phase, raising a model coupling from 32% to 73% [63]—the same reagent, and the same underlying problem, as the trazodone alkylation of Section 3.2. A heterogeneous base is a nuisance in a flask and a mechanical failure in a tube.

Solubility alone proved insufficient. At identical conditions and reactor volume, an open PFA tube (448 µL) delivered under 20%, whereas a bed packed with 60–125 µm steel spheres reached full conversion and 98% isolated yield (Scheme 8B). The packing supplies continuous mixing that segmented biphasic flow cannot, as follows: the operative design variable is the reactor geometry, not the thermostat—a lesson that returns in Section 4.2. Superheating pays a second dividend bearing on the economics of Section 3.1, since at 120 °C the reaction ran to completion at 0.15 mol% palladium, and the inexpensive XPhos matched BrettPhos.

Complementary solvent engineering was demonstrated by Yang and co-workers using *N*,*N*-dimethyloctanamide (DMO) as a cosolvent in a biphasic flow system. Their three-step synthesis of imatinib used packed-bed reactors for the piperazine substitution and subsequent Pd-catalysed C–N coupling, affording the API in 56% overall isolated yield without solvent switching or intermediate purification (Scheme 8C) [64]. The sequence is instructive for a reason unrelated to its efficiency, as follows: the palladium is reserved for the aryl–N bond to an aminopyrimidine, while the piperazine is introduced by substitution at a benzylic chloride. Even where a piperazine and a palladium catalyst appear in one route, the piperazine nitrogen is alkylated rather than arylated.

***Reductive amination and urea formation.*** Both have broad continuous precedent [65,66], including reductive amination advanced explicitly as an alternative to *N*-alkylation with alkyl halides [67], and urea synthesis used to build libraries on a piperidin-4-one scaffold [68]. These are the two disconnections at which Section 4.2 finds genuine arylpiperazine substrates, and the precedent explains why, as follows: the chemistry was established before the scaffold was attempted.

Every substrate here is a stand-in. The reaction classes underlying all three disconnections are compatible with continuous operation, and the recurring obstacles—bis-nucleophilic piperazine, precipitating base, insufficient mixing—have general recipes. What remains open is whether those recipes have been carried onto the compounds themselves.

### 4.2. Individual Flow Transformations in Drug-Specific Routes

This subsection focuses on individual continuous-flow transformations reported in routes toward cariprazine and trazodone. Three cariprazine transformations—partial ester reduction, reductive amination, and urea formation—all reported by the same industrial group, are discussed first, followed by the trazodone alkylation processes. These transformations either form bonds at disconnections 2 and 3 or prepare intermediates required for their formation. The integrated flibanserin synthesis is discussed separately in Section 4.3.

**Scheme 9:**
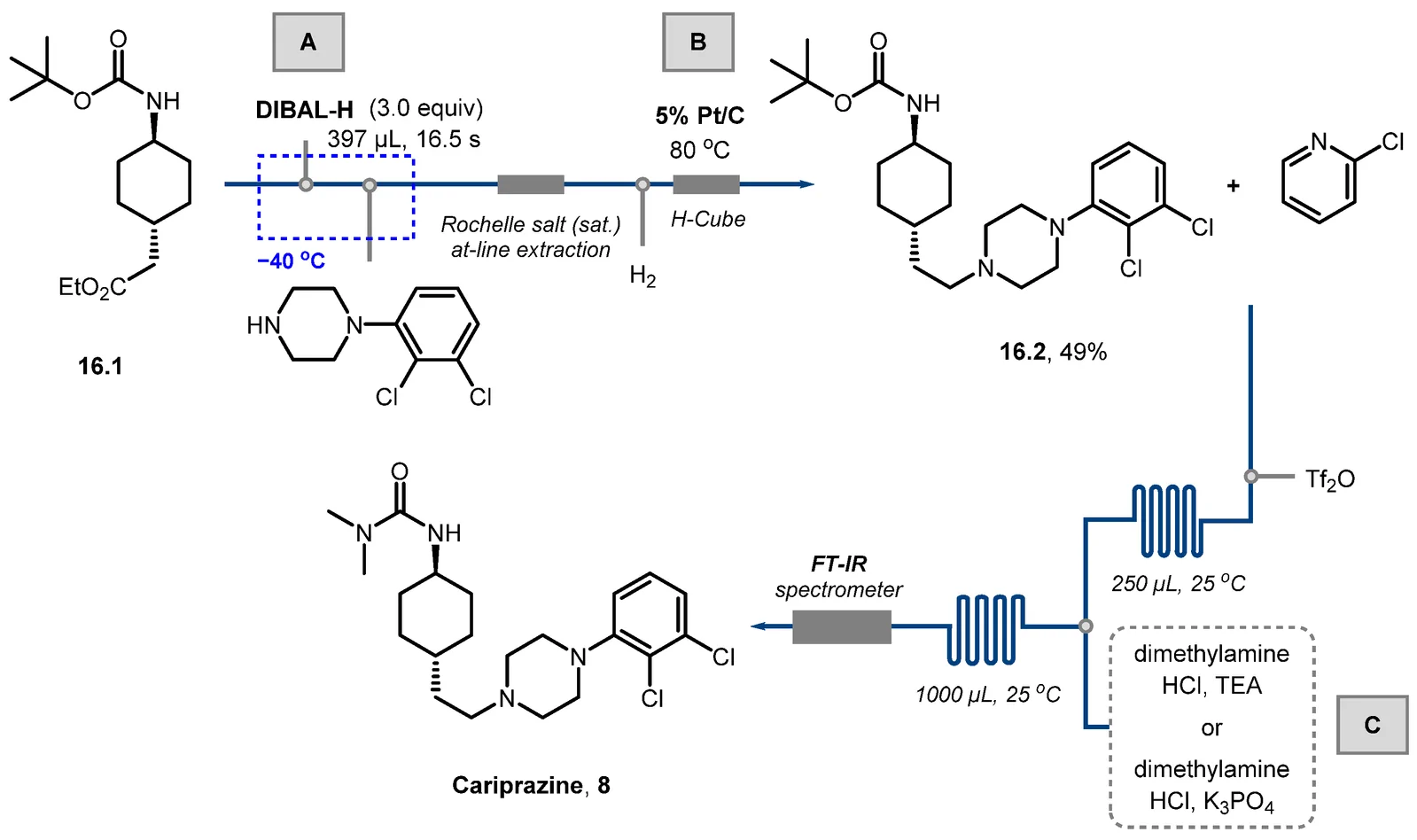
Continuous-flow steps reported on genuine cariprazine intermediates: DIBAL-H partial reduction in an alternating-diameter reactor (**A**), chemoselective reductive amination over 5% Pt/C (**B**), and urea formation via an isocyanate generated and consumed in sequence (**C**).

***A cryogenic reduction run warm.*** Partial reduction of an ester with DIBAL-H is run at −78 °C in batch to arrest over-reduction (Section 3.2). In flow the decisive variable proved to be mixing rather than temperature [25]. Plain tubing at −40 °C stalled below 30% conversion; packed beds raised it but collapsed selectivity to 52–64%. A reactor of frequently alternating internal diameter, whose repeated changes of cross-section act as mixing elements along the channel, gave 89% conversion at 98% selectivity in 16.5 s (Scheme 9). One result makes the mechanism unmistakable, as follows: within a single reactor, conversion *fell* as residence time was extended—52% at 11.4 s, 38% at 45.7 s—because the higher flow rate mixed the streams more effectively. The reaction is mixing-controlled, and the reactor geometry, not the thermostat, is the operative design choice, as the open tube and packed bed of Section 4.1 showed at identical volume.

***Catalyst and solvent as the chemoselectivity strategy.*** The resulting aldehyde was condensed with 1-(2,3-dichlorophenyl)piperazine and reduced over a packed catalyst bed, avoiding both the alkylating agent and the stoichiometric hydride. The challenge was the one anticipated in Section 2.1. A catalyst screen resolved it, as follows: 10% Pd/C gave complete conversion but 1–4% dehalogenated by-product, Raney nickel was clean but reached only 58% conversion, and 5% Pt/C at 80 °C achieved full conversion at 97% selectivity with none detected. Solvent was equally decisive—pure methanol produced substantial dehalogenation, toluene–methanol 5:1 suppressed it—and that ratio was not chosen freely, since the quench stream of the upstream module fixes it. For this cariprazine intermediate, catalyst and solvent selection was critical to chemoselectivity.

***A hazardous reagent made and consumed in place.*** At disconnection 3 the chemistry is easy and the reagent is the problem (Section 3.3), as follows: In situ generation and immediate consumption reduce hazardous-intermediate inventory, but do not eliminate the intrinsic hazards of the process. Bana and co-workers assembled non-symmetric ureas including cariprazine itself in two sequential reactors (Scheme 9C) [31]. The Boc-protected amine **16.2**—the same compound delivered by the reductions above—is treated with 2-chloropyridine and triflic anhydride in the first reactor (250 µL) to generate the isocyanate, and the stream passes directly into the second (1000 µL), where dimethylamine intercepts it. Cariprazine was obtained in 76% isolated yield using triethylamine, with residence times of 31 s and 81 s in the first and second reactors, respectively. A dimethylamine feed prepared using K_3_PO_4_ afforded an 83% isolated yield, with corresponding residence times of 31 s and 87 s. The process was monitored by in-line FTIR, and the reported productivity for the latter condition was 320 mg h^−1^.

Two features qualify claims made elsewhere. Both reactors run at ambient temperature, so the gain is neither thermal nor kinetic but purely one of inventory—the dangerous species present in milligrams rather than kilograms. Moreover, the base appears to contradict Section 4.1. The distinction is functional rather than phase-related. In the palladium coupling the base is consumed stoichiometrically and generates insoluble salts within the reacting stream; here it liberates a free amine from its hydrochloride. A practical concern is the formation of solids within the reacting stream, which may cause fouling or blockage.

***An alkylation moved for impurity control.*** The three steps above were recruited for hazard and for thermal demand. The fourth was recruited for the liability Section 3.2 identified as the most consequential at scale, as follows: the alkyl halide itself. Trazodone is assembled from a chloropropyltriazolopyridinone and 1-(3-chlorophenyl)piperazine, and residual alkylating reagents and intermediates may require assessment and control under ICH M7 [53]. Jaklova and co-workers developed a liquid–liquid phase-transfer-catalysed flow process for a chloropropyltriazolopyridinone precursor to trazodone [69]. This transformation constructs the terminal–linker bond before the arylpiperazine fragment is introduced. A separate patent describes a piperazine-first route that includes continuous formation of the chloropropyl arylpiperazine intermediate and its subsequent coupling with triazolopyridinone [70].

The patent specifies alkylating-substance limits of ≤15 ppm, with preferred limits down to <2.5 ppm. However, example-specific measurements demonstrating these impurity levels are not reported [70]. The base is again the obstacle, and again it is solved by phase transfer rather than by dissolution, as follows: a direct transfer of the solid–liquid batch conditions to continuous mode failed, and a liquid–liquid system—aqueous triazolopyridinone, potassium carbonate, and tetrabutylammonium bromide against the alkyl halide in ethyl acetate—was designed in its place [69]. The same reagent, TBAB, appears in the batch microwave alkylation of Section 3.2, in the palladium coupling of Section 4.1, and here; a heterogeneous base is a nuisance in a flask and a redesign in a tube. Each step is excellent in isolation. Section 4.3 asks what happens when they are joined.

### 4.3. Integration, Telescoping, and the Junction Problem

Three sequences have been carried beyond the single step for this scaffold. Two are described in the open literature and illustrate contrasting integration outcomes; the third, the two-reactor trazodone process noted above, is disclosed only in the patent literature and is not analysed here for want of comparable process detail. The cariprazine route required an at-line work-up between continuous modules, whereas the flibanserin route operated uninterrupted but delivered a modest overall yield.

***When the junction cannot be made continuous.*** The two reductions of Section 4.2 were designed as consecutive modules, and could not be directly connected [25]. Aluminium salts from the DIBAL-H quench precipitated on the catalyst bed and clogged it; a buffer flask, a Na_2_SO_4_·10 H_2_O drying column, and an in-line aqueous extraction all failed at the same point. A multistage at-line extraction with Rochelle salt solution enabled operation of the downstream hydrogenation module. Intermediate 16.2 was obtained in 49% overall isolated yield after chromatography and 51% after recrystallization [25]. The conversion and selectivity reported for the individual modules are analytical reaction metrics and should be distinguished from these sequence-level isolated yields.

**Scheme 10:**
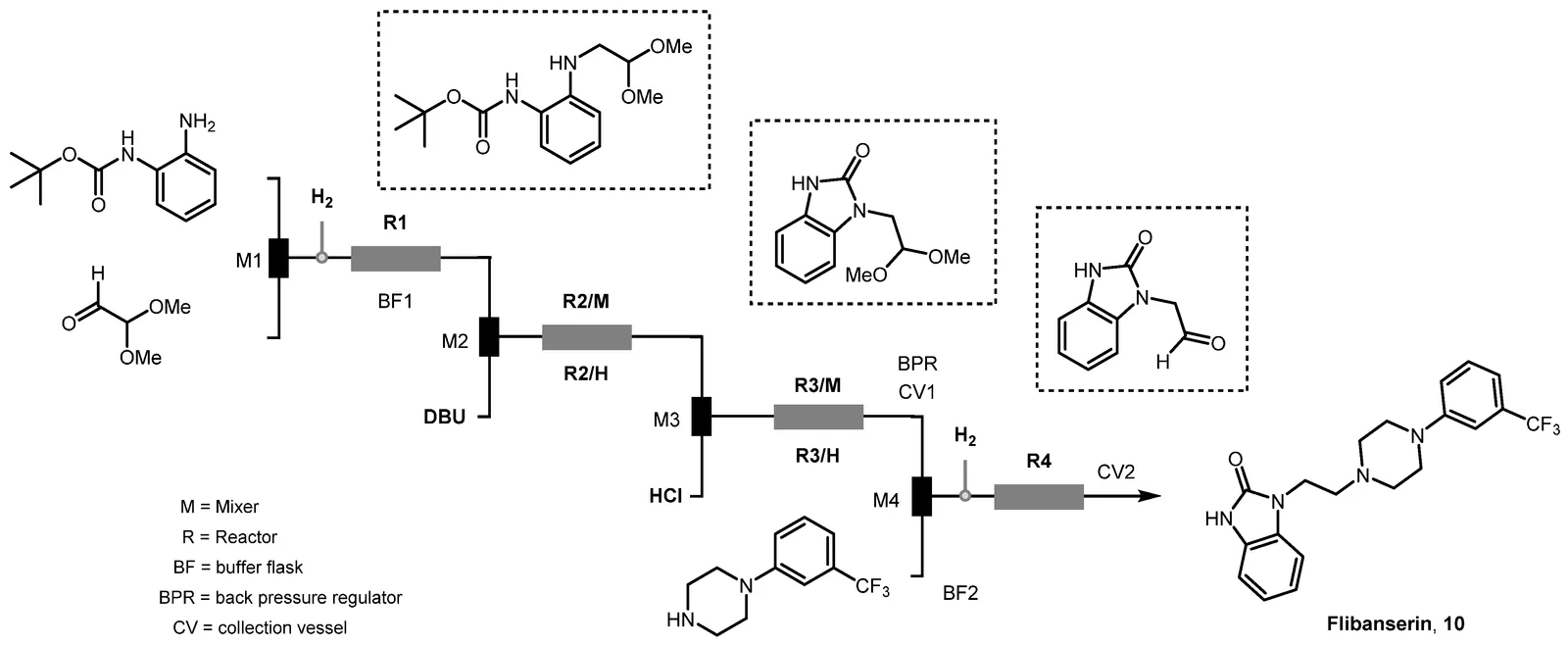
Four-step uninterrupted continuous-flow synthesis of flibanserin, comprising reductive amination, benzimidazolone ring closure, acetal hydrolysis, and a final reductive amination.

***When the route is conceived for the medium.*** The four-step, uninterrupted synthesis of flibanserin reported by Bana and co-workers is the most complete demonstration to date (Scheme 10) [20]. Its significance lies less in any single step than in the fact that it was not a transcription of an existing batch process. Rather than adapt the medicinal-chemistry sequence—which builds the benzimidazolone core through protection, selective alkylation, and stoichiometric base—the authors redesigned the disconnections for continuous operation. Two reductive aminations over 10% Pd/C bracket the sequence at 100 °C and 10 bar, with residence times of roughly 8 and 12 s; between them, the Boc group is not removed as waste but consumed as the carbonyl of the benzimidazolone, closed with DBU at 200 °C in 7.4 min; and an HCl-mediated acetal hydrolysis is integrated with a biphasic separation. Gas–liquid and liquid–liquid separators couple the steps and strip excess reagents, so the system reached steady state and ran for over 2.5 h without manual intervention. The total residence time was below 20 min, and flibanserin was isolated as its hydrochloride in 31% overall isolated yield after off-line salt formation and crystallization [20].

The reported flow route used DBU in place of NaH or KO*^t^*Bu to address solubility constraints. During hydrolysis, product partitioning between the phases was addressed by setting the aqueous-to-organic ratio at 1:10 and providing mixing with a sand-packed column [20]. These process adjustments illustrate the importance of inter-step compatibility in flow-oriented design [20,71].

***The two sequences illustrate different integration requirements.*** The cariprazine route uses an at-line extraction between continuous modules, whereas the flibanserin route links four reaction steps through in-line separations. Because the reported yields refer to different products and sequences, they do not support a direct comparison of process performance [20,25].

These two cases illustrate benefits that continuous processing can offer beyond yield. A hazardous intermediate can be generated and consumed without ever accumulating; a protecting group can be made a building block rather than waste; intermediate isolation can be dispensed with entirely; and production can be increased by extending operating time, subject to catalyst lifetime, fouling, steady-state stability, and process-control requirements. Realizing these benefits requires appropriate route and process design. Telescoping is therefore less an exercise in chemistry than in interface engineering.

### 4.4. Coverage: What Flow Has and Has Not Touched

**Figure 3:**
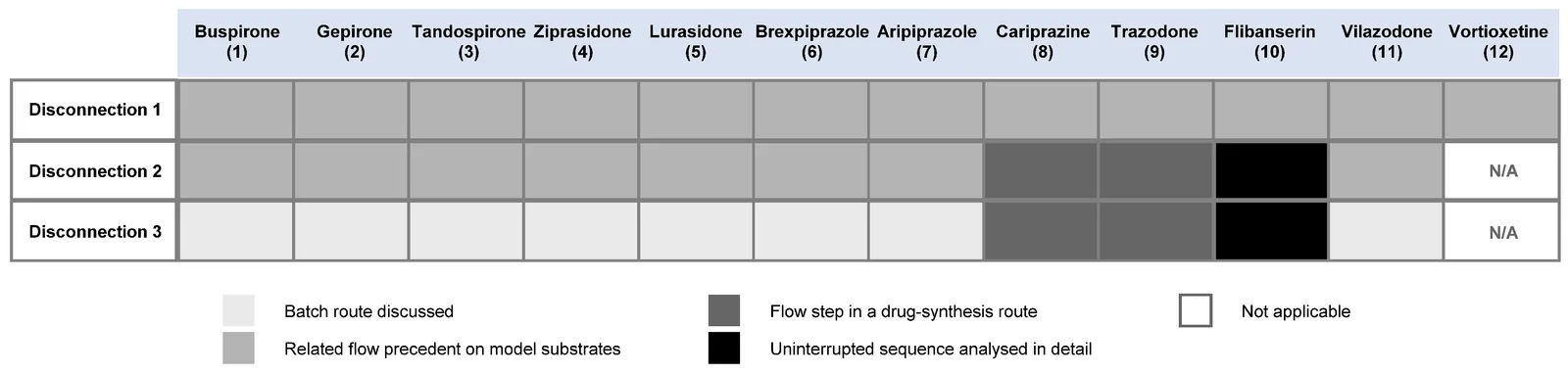
Coverage of the three disconnections by the batch and continuous-flow routes examined in this review. Light grey indicates batch routes discussed in Section 3 for which no corresponding drug-specific flow example was identified among the reports reviewed. Medium grey indicates related flow precedents on model substrates, without validation on the listed drug (Disconnection 1: [62,63,64]; Disconnection 2: [65,66,67]). Dark grey indicates drug-specific flow transformations: cariprazine at Disconnection 2 [25] and Disconnection 3 [31], and trazodone at Disconnection 2 [70] and Disconnection 3 [69,70]. Black indicates transformations within the uninterrupted flibanserin reaction sequence at Disconnections 2 and 3 [20]. White cells marked N/A indicate disconnections not applicable to the scaffold. Patent-only sequences are represented at the individual-transformation level.

Read back onto the three disconnections, the material of Section 4.1, Section 4.2 and Section 4.3 forms a sparse and highly patterned map (Figure 3).

Disconnection 1 has no drug-specific flow example among the reports examined here. Continuous SNAr and palladium-catalysed C–N coupling have been demonstrated on model substrates (Section 4.1), but these examples do not establish preparation of the aryl–N bond in the agents listed in Table 1. The corresponding arylpiperazine building blocks are introduced pre-formed in the drug-directed flow routes reviewed here.

Disconnection 2 is represented by the cariprazine reductive amination and the trazodone *N*-alkylation processes discussed in Section 4.2, and by the final reductive amination in the integrated flibanserin sequence (Section 4.3). The DIBAL-H ester reduction in the cariprazine route is a supporting upstream transformation, as follows: it generates the aldehyde required for bond formation rather than forming the piperazine–linker bond itself.

Disconnection 3 is represented by cariprazine urea formation and by terminal–linker *N*-alkylation in trazodone routes. In the integrated flibanserin sequence, the first reductive amination forms the C–N bond that becomes the linker–terminal connection after benzimidazolone ring closure. For the imide-forming or imide-alkylation routes to buspirone, gepirone, tandospirone, and lurasidone, and the Friedel–Crafts acylation–reduction route to vilazodone, no corresponding drug-specific flow implementation is identified among the reports reviewed here.

The reported implementations cluster around identifiable process constraints rather than covering all three disconnections evenly. The DIBAL-H reduction addresses low-temperature operation, catalytic reductive amination requires chemoselectivity control, in situ isocyanate generation limits hazardous-intermediate inventory, and the trazodone processes address alkylation and impurity control requirements. These constraints overlap the hazard, waste, energy, and impurity control categories of Section 3.4, but do not correspond to them on a one-to-one basis.

This distribution supports a selective rather than comprehensive approach to flow implementation. However, the absence of a published flow example does not establish that batch is intrinsically preferable for that transformation. Section 5 translates the reported cases into practical selection criteria, while distinguishing a specific process benefit from the operational case for repeated continuous production.

## 5. Deciding What to Move into Flow

This section proposes practical criteria for evaluating batch and continuous-flow options based on the cases reviewed. Their relevance depends on the substrate, production requirements, and process conditions; they should not be interpreted as universal selection rules.

### 5.1. Triggers and Practical Limitations

Section 3.4 identifies four categories of batch-route liability, as follows: hazard, waste, energy demand, and impurity control. These categories are not a one-to-one list of flow-selection triggers. The cases reviewed here instead suggest three technical triggers—demanding thermal or mixing conditions, hazardous-reagent or intermediate inventory, and stringent selectivity or impurity-control requirements—together with an operational consideration, as follows: repeated production of a fixed substrate (Table 2).

- **Demanding thermal or mixing conditions.** The cariprazine DIBAL-H example illustrates how improved mixing enabled operation at −40 °C rather than the conventional −78 °C. Superheated substitutions provide a separate example of reaction intensification in flow (Section 4.1 and Section 4.2).
- **Hazardous reagent or intermediate inventory.** In the cariprazine urea synthesis, generation and immediate consumption of the isocyanate reduce the need to isolate or store the reactive intermediate (Section 4.2).
- **Stringent selectivity or impurity-control requirements.** Catalyst and solvent selection suppress dehalogenation in the cariprazine reductive amination, while the trazodone processes address the handling and control of alkylating species (Section 4.2).
- **Repeated production of a fixed substrate.** Repeated demand strengthens the practical case for developing and maintaining a continuous process. The uninterrupted flibanserin synthesis illustrates integrated operation (Section 4.3), but repetition alone does not establish an advantage over batch.

Waste reduction is evaluated across the complete route rather than treated as an automatic consequence of continuous operation. The following practical limitations should also be considered when evaluating flow implementation.

- **Solids formation.** Salt precipitation within the reacting stream can cause fouling or blockage. The significance of this limitation depends on the reactor and operating conditions (Section 4.1 and Section 4.2).
- **Substrate diversity.** Changes in substrate structure may require reoptimization of reaction and operating conditions. Their effect on the practicality of flow should be assessed for the intended analogue series.
- **Setup and changeover time.** For small-scale analogue synthesis, setup, stabilization, and cleaning requirements may offset the benefits of flow and should be compared with the requirements of parallel batch processing.
- **Commercial availability of building blocks.** No drug-specific continuous-flow construction of the aryl-*N*(piperazine) bond was identified among the reports reviewed here. Commercial availability of the corresponding arylpiperazine fragments may reduce the immediate incentive to develop their synthesis in-house (Section 2.2 and Section 4.4)

### 5.2. Judging the Process, Not the Reaction

The multistep examples in Section 4.3 show that the performance of an individual flow step does not establish the benefit of an integrated process. Inter-step compatibility therefore requires evaluation across the sequence. Where direct integration is impractical, a hybrid arrangement combining continuous modules with intermediate work-up may be appropriate, but its overall performance must be assessed.

### 5.3. From Discovery to Manufacture, with One Caution for This Class

During early-stage analogue synthesis, the choice between batch and flow should account for substrate diversity, setup and changeover requirements, and opportunities for parallel processing. As development focuses on a defined intermediate or candidate, repeated demand may justify evaluating flow to address an identified process limitation.

In the cariprazine reductive-amination study, dechlorination depended on catalyst and solvent selection [25]. This finding supports evaluating halogen retention for the specific substrate and reduction conditions; it does not establish a common limitation for all halogenated or trifluoromethylated arylpiperazines.

## 6. Conclusions

This review has examined batch and continuous-flow approaches to arylpiperazine-based CNS ligands in terms of three principal synthetic disconnections. Reading the flow literature by degree of implementation rather than by bond produces a sparse map, distinguishing reaction-class demonstrations on model substrates from individual transformations in drug-directed routes and multistep sequences. The individual transformations discussed focus on routes toward cariprazine and trazodone. Two open-literature multistep sequences are analysed in detail, as follows: the route toward a cariprazine intermediate, which requires an at-line work-up between continuous modules, and the uninterrupted four-step synthesis of flibanserin. A further multistep trazodone sequence is disclosed in the patent literature and is discussed here at the individual-transformation level.

This narrative review has several limitations. Literature selection may introduce selection bias, and drug-specific flow examples are sparse within the reviewed literature. The key cariprazine flow studies reviewed here originate from a single research group [25,31]. Heterogeneous reporting of reaction outcomes and process metrics limits direct comparisons between studies. Comparable economic and environmental assessments and long-duration operating data are also limited within this evidence base. The decision framework in Section 5 therefore represents the authors’ interpretation of the reported cases, and its general applicability has not been validated.

The reviewed examples show flow implementations addressing selected safety, mixing, chemoselectivity, and impurity-control challenges. No drug-specific flow formation of the aryl-nitrogen bond was identified within the reviewed literature; the drug-directed flow routes examined use preformed arylpiperazine fragments. This distribution does not establish that transformations lacking reviewed flow examples are intrinsically unsuitable for continuous processing.

The cariprazine sequence required an at-line work-up between continuous modules, whereas the flibanserin sequence achieved uninterrupted four-step operation. These examples highlight the importance of solvent, reagent, work-up, and operating-condition compatibility when connecting continuous modules [20,25].

Potential research directions include catalyst and base systems that limit precipitation during continuous C–N coupling [63,64], catalysts and conditions that improve halogen retention in susceptible substrates, and the use of in-line analytics with automated optimization. These opportunities require evaluation for individual arylpiperazine routes. The proposed framework connects synthetic disconnections, implementation level, and inter-step compatibility to support that evaluation.

## Figures and Tables

**Table 1:** Representative CNS-active drugs containing the 1-arylpiperazine motif.

Number	Drug, Ref.	Aryl/heteroaryl Group, Linker	Principal Targets, Indication	Structure
1	Buspirone [6]	Pyrimidin-2-yl, Butyl–azaspirodecanedione	5-HT1A partial agonist Anxiety	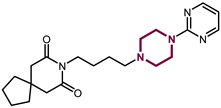
2	Gepirone [26]	Pyrimidin-2-yl, Butyl–dimethylglutarimide	5-HT1A partial agonist, MDD	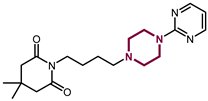
3	Tandospirone [27]	Pyrimidin-2-yl, Butyl–norbornane dicarboximide	5-HT1A partial agonist Anxiety, depression	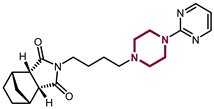
4	Ziprasidone [7]	Benzisothiazol-3-yl, Ethyl–oxindole	D2; 5-HT2A Schizophrenia	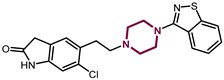
5	Lurasidone [28]	Benzisothiazol-3-yl, *trans*-Cyclohexanedimethyl–norbornane imide	D2, 5-HT2A, 5-HT7 antagonist; 5-HT1A partial agonist Schizophrenia, bipolar depression	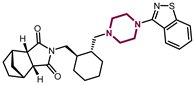
6	Brexpiprazole [29]	Benzothiophen-4-yl, Butoxy–quinolinone	D2 partial agonist; 5-HT1A partial agonist, 5-HT2A antagonist Schizophrenia, MDD (adjunct)	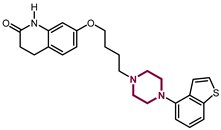
7	Aripiprazole [8]	2,3-Dichlorophenyl, Butoxy–quinolinone	D2 partial agonist; 5-HT1A, Schizophrenia	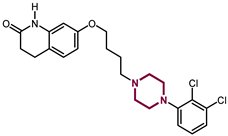
8	Cariprazine [9]	2,3-Dichlorophenyl, Ethyl–cyclohexyl urea	D3/D2 partial agonist Schizophrenia, bipolar I	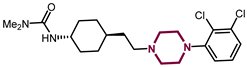
9	Trazodone [10]	3-Chlorophenyl, Propyl–triazolopyridinone	5-HT2A; SERT Depression	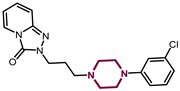
10	Flibanserin [11]	3-(Trifluoromethyl)phenyl, Ethyl–benzimidazolone	5-HT1A agonist; 5-HT2A antagonist HSDD	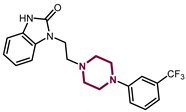
11	Vilazodone [12]	2-Carbamoylbenzofuran-5-yl, Butyl-5-cyanoindol-3-yl	SERT; 5-HT1A partial agonist Depression	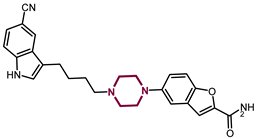
12	Vortioxetine [13]	2-(2,4-Dimethylphenylthio)phenyl, Direct (no linker)	SERT; 5-HT1A, 5-HT3, 5-HT7 Depression	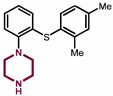

Abbreviations: 5-HT, 5-hydroxytryptamine; SERT, serotonin transporter; MDD, major depressive disorder; HSDD, hypoactive sexual desire disorder.

**Table 2:** Transformations of arylpiperazine synthesis assessed against the triggers and practical limitations of Section 5.1, with the level of continuous implementation reported for this scaffold.

Transformation	Typical Batch Conditions	Flow Implementation	Trigger	Practical Limitation	Level Reported	Ref.
SNAr (activated azine)	Piperazine, K_2_CO_3_/DIPEA, MeCN or DMF, 80–140 °C, 12–24 h	Superheated coil above solvent bp with back-pressure regulator, min-scale residence	Thermal extreme (hours of reflux)	Bis-nucleophilic piperazine → excess amine or added module; salt precipitation	Model only	[62]
Pd-catalysed C–N coupling	Pd_2_(dba)_3_/BINAP, NaO*^t^*Bu, toluene, 80 °C to reflux	Packed-bed reactors with aqueous base; phase-transfer catalyst or DMO cosolvent	Repeated delivery; low Pd loading at 120 °C	Base consumed stoichiometrically → salts deposit in the stream	Model only	[63,64]
Terminal-linker *N*-alkylation (trazodone precursor)	K_2_CO_3_/TBAB, MeCN, 50 °C, ultrasound (solid-liquid system)	Liquid-liquid phase-transfer Catalysis before arylpiperazine introduction Approximately 45% HPLC yield (combined chloropropyl and bromopropyl precursors); 50 °C; residence time 60 min; pulsed ultrasound	Interphase mass-transfer and mixing requirements	Solid–liquid batch conditions required reformulation for flow	Drug-specific precursor transformation	[69] (journal article)
*N*-Alkylation in a piperazine-first trazodone route	Alkali metal carbonate, catalytic iodide, DMF or DMAc (terminal coupling; cited batch precedent)	Flow preparation of the chloropropyl arylpiperazine intermediate and subsequent coupling with triazolopyridinone Terminal coupling (Example 30): 66% isolated yield of trazodone base; 145 °C; residence time 120 s. Throughput and operating duration not reported for this example.	Impurity-control objective; patent-specified limits	Off-line work-up and batch salt formation in the reported examples	Drug-specific transformations; patent disclosure	[70] (patent)
Reductive amination	Aldehyde + arylpiperazine, NaBH(OAc)_3_, DCE, rt	Packed-bed catalytic hydrogenation, 5% Pt/C, 80 °C, toluene–MeOH 5:1 Full conversion; 97% selectivity	Selectivity held (hydrodehalogenation); no stoichiometric hydride	Solvent ratio fixed by the upstream quench	Genuine intermediate	[25]
Ester → aldehyde (DIBAL-H)	−78 °C, cryogenic, over-reduction risk	Alternating-diameter micro-reactor, −40 °C, 16.5 s 89% conversion; 98% selectivity; Residence time 16.5 s	Thermal extreme; mixing-controlled	Al salts precipitate → at-line extraction before catalyst bed	Genuine intermediate	[25]
Urea formation	Isocyanate or carbamoyl chloride, rt–60 °C	Two sequential reactors, in-line FT-IR, isocyanate generated in situ 83% isolated yield; Residence times 31 s and 87 s; Throughput 320 mg h^−1^	Hazardous reagent (inventory)	Setup and changeover requirements for analogue synthesis	Drug-specific transformation	[31]
Imide/amide terminal	HATU or acid chloride, DMF, rt; imide alkylation, K_2_CO_3_, DMF, 125–140 °C	—	—	—	Not identified in this review	—
Multistep telescoping	Isolate each intermediate	Gas–liquid and liquid–liquid separators, steady state (flibanserin, 4 steps) 31% overall isolated yield; Total residence time < 20 min; operating duration > 2.5 h Off-line salt formation and crystallization.	Repeated delivery; no intermediate isolation	Route adaptation may be required; solvent/phase compatibility between steps	Integrated	[20]

## Data Availability

No new data were created or analyzed in this study. Data sharing is not applicable to this article.
